# 3PM-guided innovation in treatments of severe alcohol-associated hepatitis utilizing fecal microbiota transplantation

**DOI:** 10.1007/s13167-024-00381-5

**Published:** 2024-10-31

**Authors:** Lubomir Skladany, Natalia Kubanek, Svetlana Adamcova Selcanova, Daniela Zilincanova, Daniel Havaj, Karolina Sulejova, Katarina Soltys, Lucia Messingerova, Michal Lichvar, Lukas Laffers, Michal Zilincan, Eva Honsova, Peter Liptak, Peter Banovcin, Jan Bures, Tomas Koller, Olga Golubnitschaja, Juan-Pablo Arab

**Affiliations:** 1 HEGITO - Department of Hepatology, Gastroenterology and Liver Transplantation of F. D., Roosevelt University Hospital, Banska Bystrica, Slovakia; 2 Department of Microbiology and Virology, Faculty of Natural Sciences, Comenius University Bratislava, Bratislava, Slovakia; 3 Centre of Biosciences, Institute of Molecular Physiology and Genetics, Slovak Academy of Sciences, Bratislava, Slovakia; 4 Faculty of Chemical and Food Technology, Institute of Biochemistry and Microbiology, Slovak University of Technology, Bratislava, Slovakia; 5 Geneton Ltd., Bratislava, Slovakia; 6 Department of Mathematics, Faculty of Natural Sciences, Matej Bel University, Banska Bystrica, Slovakia; 7 Department of Radiology, FD Roosevelt Faculty Hospital, Banska Bystrica, Slovakia; 8 UniLabs S.R.O - Pathology, Prague, Czech Republic; 9 Jessenius Faculty of Medicine in Martin (JFM CU), Gastroenterology Clinic JFM CU, Comenius University in Bratislava, Martin, Slovakia; 10 Department of Internal Medicine, Charles University First Faculty of Medicine and Military University Hospital Prague, Prague, Czech Republic; 11 Institute of Gastrointestinal Oncology, Military University Hospital Prague, Prague, Czech Republic; 12 Gastroenterology and Hepatology Subdivision, 5Th Department of Internal Medicine, Comenius University Faculty of Medicine, University Hospital Bratislava, Bratislava, Slovakia; 13 Predictive, Preventive and Personalised (3P) Medicine, University Hospital Bonn, Rheinische Friedrich-Wilhelms-Universität Bonn, 53127 Bonn, Germany; 14 Division of Gastroenterology, Department of Medicine, Schulich School of Medicine, Western University & London Health Sciences Centre, London, ON Canada; 15 Departamento de Gastroenterologia, Escuela de Medicina, Pontificia Universidad Catolica de Chile, Santiago, Chile

**Keywords:** Severe alcohol-associated hepatitis, Alcohol toxicity, Survival, Predictive preventive personalized medicine (PPPM / 3PM), Gut microbiota, Dysbiosis, Systemic inflammation, Mitochondrial health, Fecal microbiota transplantation, Multi-level diagnostics, Patient stratification, Phenotyping, Individualized patient profile, Tailored therapy, Cost-efficacy, Health policy

## Abstract

**Rationale:**

Severe alcohol-associated hepatitis (SAH) is the most critical, acute, inflammatory phenotype within the alcohol-associated liver disease (ALD) spectrum, characterized by high 30- and 90-day mortality. Since several decades, corticosteroids (CS) are the only approved pharmacotherapy offering highly limited survival benefits. Contextually, there is an evident demand for 3PM innovation in the area meeting patients’ needs and improving individual outcomes. Fecal microbiota transplantation (FMT) has emerged as one of the new potential therapeutic options. In this study, we aimed to address the crucial 3PM domains in order to assess (i) the impact of FMT on mortality in SAH patients beyond CS, (ii) to identify factors associated with the outcome to be improved (iii) the prediction of futility, (iv) prevention of suboptimal individual outcomes linked to increased mortality, and (v) personalized allocation of therapy.

**Methods:**

We conducted a prospective study (NCT04758806) in adult patients with SAH who were non-responders (NR) to or non-eligible (NE) for CS between January 2018 and August 2022. The intervention consisted of five 100 ml of FMT, prepared from 30 g stool from an unrelated healthy donor and frozen at − 80 °C, administered daily to the upper gastrointestinal (GI) tract. We evaluated the impact of FMT on 30- and 90-day mortality which we compared to the control group selected by the propensity score matching and treated by the standard of care; the control group was derived from the RH7 registry of patients hospitalized at the liver unit (NCT04767945). We have also scrutinized the FMT outcome against established and potential prognostic factors for SAH — such as the model for end-stage liver disease (MELD), Maddrey Discriminant Function (MDF), acute-on-chronic liver failure (ACLF), Liver Frailty Index (LFI), hepatic venous-portal pressure gradient (HVPG) and Alcoholic Hepatitis Histologic Score (AHHS) — to see if the 3PM method assigns them a new dimension in predicting response to therapy, prevention of suboptimal individual outcomes, and personalized patient management.

**Results:**

We enrolled 44 patients with SAH (NR or NE) on an intention-to-treat basis; we analyzed 33 patients per protocol for associated factors (after an additional 11 being excluded for receiving less than 5 doses of FMT), and 31 patients by propensity score matching for corresponding individual outcomes, respectively. The mean age was 49.6 years, 11 patients (33.3%) were females. The median MELD score was 29, and ACLF of any degree had 27 patients (81.8%). FMT improved 30-day mortality (*p* = 0.0204) and non-significantly improved 90-day mortality (*p* = 0.4386). Univariate analysis identified MELD ≥ 30, MDF ≥ 90, and ACLF grade > 1 as significant predictors of 30-day mortality, (*p* = 0.031; *p* = 0.014; *p* = 0.034). Survival was not associated with baseline LFI, HVPG, or AHHS.

**Conclusions and recommendations in the framework of 3PM:**

In the most difficult-to-treat sub-cohort of patients with SAH (i.e., NR/NE), FMT improved 30-day mortality. Factors associated with benefit included MELD ≤ 30, MDF ≤ 90, and ACLF < 2. These results support the potential of gut microbiome as a therapeutic target in the context of 3PM research and vice versa — to use 3PM methodology as the expedient unifying template for microbiome research. The results allow for immediate impact on the innovative concepts of (i) *personalized phenotyping and stratification of the disease* for the clinical research and practice, (ii) *multilevel predictive diagnosis* related to personalized/precise treatment allocation including evidence-based (ii) *prevention of futile and sub-optimally effective therapy*, as well as (iii) *targeted prevention* of poor individual outcomes in patients with SAH. Moreover, our results add to the existing evidence with the potential to generate new research along the SAH’s pathogenetic pathways such as diverse individual susceptibility to alcohol toxicity, host-specific mitochondrial function and systemic inflammation, and the role of gut dysbiosis thereof.

**Supplementary Information:**

The online version contains supplementary material available at 10.1007/s13167-024-00381-5.

## Introduction

Severe alcohol-associated hepatitis is taking an increasing global toll on ever younger lives [1–7], and the dismal trend is predicted to progress [2, 6, 8–11]. Effective therapeutic modalities for SAH beyond abstinence, nutrition, antimicrobials, and CS — the only approved pharmacotherapy since the 1970s — are unmet needs [12–21]. As around half the patients with SAH do not qualify for CS due to contraindications or do not respond to them, a cohort of patients “beyond CS” has been burgeoning and is paralleled by increasing pressure on 3PM; 3PM’s response is evolving along the two avenues — intensifying the search for new experimental therapeutic options and, optimizing existing bundle of SAH care [22–27]. Since Central Europe is the world’s hot spot for liver cirrhosis and ALD, we set out to search the pipeline of experimental therapies for modalities that would match both — the region-specific healthcare milieu and the 3PM research template [28, 29]. Fecal microbiota transplantation has arisen as the ideal candidate since it merged well-grounded pathophysiological theory, promising results of landmark trials, reassuring safety signals, and permissive ethical, economic, and logistical demands [30–36].

In this study, we aimed to investigate the help of the 3PM template in the treatment with FMT of patients with SAH beyond CS; the juxtaposition of the template, the disease, and the therapy brought about four areas of scrutiny, each with three domains, and resulted in the working hypothesis: 1. 3PM *vs.* SAH. *P1. Predictive domain.* On top of existing research on the prediction of poor outcomes (using clinical factors such as MELD, MDF, and ACLF grades), there exists continuing new research developing predictive tools to stratify patients for tailored experimental therapeutic targets such as gut dysbiosis, mitochondrial health, and many others. *P2. The preventive domain* focuses on implementing strategies to prevent disease progression and death through early and effective intervention. This domain overlaps with P3 in that it prevents futile research and loss of resources. *P3. Personalized medicine.* Research on FMT in SAH will enable us to tailor treatment protocols to individual patient profiles based on pre-treatment microbiome and clinical characteristics [12–27, 30–36]. 2. 3PM and gut dysbiosis in SAH. *P1.* Analyzing gut microbial patterns pre- and post-FMT in terms of the prediction of disease severity and response to treatment with FMT will enable us to forecast individual patient outcomes and tailor treatment plans [37]. Results will also set the stage for preventing disease-specific gut dysbiosis through lifestyle and dietary interventions, probiotics, and other microbiome-directed approaches. Early personalized intervention targeting dysbiosis can prevent disease progression and poor individual outcomes. *P2/P3.* Profiling microbiomes to identify specific dysbiosis patterns will enable individualized treatment; and, by tailoring FMT based on the patient’s unique microbiome composition and clinical characteristics, prevent entering blind-ended therapeutic and research arms. 3. 3PM *vs.* FMT. *P1.* To predict FMT effectiveness by analyzing pre-treatment microbiome profiles and identifying biomarkers of success/therapeutic targets. Select suitable candidates for FMT. *P2.* Use FMT to restore healthy microbiome and prevent recurrent infections, resistance to antibiotics, leaky gut with low-grade inflammation, as well as disease recurrence and complications. *P2/3.* Select for FMT donor microbiota that best matches the recipient’s needs. Consider donor-recipient microbiome compatibility and specific beneficial microbial strains for individualized treatment [32–36, 38–43]. 4. 3PM and FMT in SAH. *P1.* Identify SAH NR/NE patients likely to benefit from FMT using predictive factors such as MELD scores, MDF, and ACLF grades, as well as pre-FMT microbiome analysis. Direct FMT to patients with a high likelihood of positive response. *P2.* Prevent disease progression by restoring gut microbiota balance, and gut barrier function, reduce low-grade systemic inflammation, and improve liver function. *P3.* Tailoring FMT treatment to individual patient profiles, considering disease severity by clinical characteristics, and pre-treatment microbiome composition. Our data will help administer FMT to patients with the highest likelihood of benefit while exploring alternative treatments for others [32–36, 38–43]. 5. Working hypothesis questions. 5. (a) Individualized patient profiling and stratification. Our prospective study (NCT04758806) aims at answering the following questions:

1. Are there pre-treatment (baseline) patient characteristics that would allow for multilevel predictive diagnostics, personalized SAH phenotyping, and patient stratification, which would help to ensure targeted prevention of both poor individual outcomes and suboptimally effective therapy? Which of the following pre-treatment variables can be used for 3 PM purposes and clinical practice?
  - Microbiome analysis [37]
  - Demographic characteristics—age and gender
  - The severity of SAH according to MDF and MELD score
  - Presence/absence of ACLF and its grades
  - Frailty by the LFI
  - Portal hypertension by HVPG
  - Liver histology was obtained via transjugular biopsy and examined by one investigator (EH)
2. Are there FMT-related variables determining treatment response and post-treatment course of the disease (comparison to literature) [44]
3. Will FMT improve the results of the guideline-recommended endpoints against which the 3PM domains are to be challenged [24]
  - 30—day mortality
  - 90 – day mortality

5. (b) Systemic inflammation. Alcohol abuse triggers inflammation through PAMPs (pathogen-associated molecular patterns) and DAMPs (damage-associated molecular pattern molecules), which are recognized by pattern-recognition receptors (PRRs) such as Toll-like receptors (TLRs) [45–49]. PAMPs from microorganisms reach the liver via the lymphatic system and portal circulation, with lipopolysaccharide (LPS) from Gram-negative bacteria being a significant TLR stimulator. Chronic alcohol use increases intestinal permeability leading to a so-called leaky gut and facilitates PAMP translocation; this leads to gut-liver axis signals activating Kupffer and other immune cells, activating canonical and non-canonical inflammasome cascades and leading to cytokine production, including TNF (Tumor necrosis factor) and IL-6 (Interleukin 6); these molecules are linked to poor individual outcomes in acute alcohol-associated hepatitis (AH). The AH-associated systemic inflammation also includes DAMPs like ATP (Adenosine triphosphate), DNA (Deoxyribonucleic acid), and uric acid which can be elevated in the blood [45, 50, 51]. Alcohol promotes cell death through mitochondrial apoptosis and endoplasmic reticulum stress [52, 53] [54–56]. This can lead to systemic inflammatory response syndrome (SIRS) and sepsis-like syndrome which is difficult to distinguish from Gram-negative bacterial infections-induced SIRS and sepsis [57, 58]. 5. (c) Microbiome, inflammation, and mitochondrial health. The gastrointestinal tract hosts over a trillion microorganisms — more than human cells with incomparably more genes than human genome [59]. Gut microbiota comprises bacteria, viruses, fungi, protozoa, and archaea, whereas gut microbiome is represented by the genetic material of microbiota together with the so-called theater of activity of the microbiota [60, 61]. Gut microbiota assists digestion, metabolism, and immunity and can produce alcohol endogenously. The gut-liver connection involves a.o. microorganisms and their products reach the liver via lymphatics and portal circulation, while bile from the liver is secreted into the intestine and regulates the microbiome [62, 63]. Alcohol consumption considerably alters the gut microbiome by increasing harmful Gram-negative bacteria, decreasing commensals, especially SCFA-producing strains, shifting mycobiome and virome, and compromising the intestinal barrier [35, 64–70]. Alcohol-associated dysbiosis also alters bile acid metabolism, increasing toxic secondary bile acids and reducing protective primary bile acids, leading to gut and liver damage. Moreover, in ALD, small intestinal bacterial overgrowth (SIBO) and reduced *Lactobacillus* species are common, leading to decreased bactericidal substance production and compromised gut homeostasis [71]. Of interest, gut dysbiosis in cirrhosis bears resemblance to the oral microbiome (so-called oralization of the gut microbiome) which underscores the importance of oral health in liver diseases [72–74]. All these pathological processes lead to dysbiosis-mediated systemic inflammation which is one of the main drivers of ALD and its progression; in the case of SAH, these processes are accentuated to the extreme [60, 61]. As in the majority of patients SAH is associated with ACLF, it is of interest to investigate not only the link between gut dysbiosis and inflammatory response but also to see the association of dysbiosis with the second most important pathogenetic cascade of ACLF — deranged mitochondrial health [45, 75, 76]. 5. (d) Targeted prevention of health risks and improved individual outcomes. Targeted prevention of health risks, one of the cornerstones of the 3PM concept, spans in SAH the wide spectrum of possible actions from tackling alcogenicity of the society by the political and public health actions on the left side of the spectrum, to the prevention of mortality by targeting microbiome and mitochondrial health on the right side [1]. As 30-day mortality of SAH in NR to CS can be as high as 35–50%, in this paper, we focus on the potential of FMT to prevent this health risk [77]. As mentioned above, using FMT in SAH can be considered an example of targeted prevention of poor individual outcomes because it leverages the principal pathophysiological cascade operative in the disease [60, 61, 78, 79]. In this line, several studies have addressed SAH by FMT for improved individual outcomes with promising 30-day to 3-year outcomes [80–82]. Moreover, targeting the microbiome proved effective in improving also patient-reported outcomes mediated by hepatic encephalopathy and alcohol craving [41, 42, 72].

## Patients’ recruitment and methodology

### Study design

In this prospective study (NCT04758806), we enrolled adult patients with SAH who were either CS-NR or CS-NE, between January 2018 and August 2022 [24, 83]. The study was conducted at the academic liver and transplant unit. Ethical approval was obtained from the institutional review board, and all participants provided informed consent.

### Patient’s recruitment

Patients were eligible for inclusion if they met the following criteria: Patients were diagnosed with SAH based on NIAAA criteria, i.e., with chronic (years) and recent (weeks) heavy alcohol abuse (> 50–60 g/day), with sudden onset of jaundice, with elevated aspartate aminotransferase (AST) above the upper limit of normal range, AST:ALT (alanine aminotransferase) ratio > 1.5, AST and ALT < 400 IU/mL, and with excluded other etiologies of acute hepatitis. Transjugular liver biopsy (TJB) with simultaneous HVPG measurement has been indicated whenever possible/necessary. We included adults (> 18 years of age) with SAH NR according to Lille criteria at day 7 or NE due to CS contraindications such as active infection or gastrointestinal bleeding [13, 18]. The control group of SAH NR/NE patients who were not treated with FMT but with the current guideline-recommended standard of care had been selected from the cirrhosis registry RH7 (NCT04767945) by the propensity-score matching as described below [26]. We excluded patients with malignancy and organ failures outside the context of ACLF as diagnosed by CANONIC—EASL/EF CLIF criteria [84], severe uncontrolled psychiatric syndromes except for hepatic encephalopathy, non-compliance with the study protocol, or withdrawal of consent [85].

#### Recorded variables

We recorded demographics, severity of SAH by MELD, MDF, and ACLF, inflammatory markers, frailty by LFI, portal pressure by HVPG, and histology by AHHS. The exposure was FMT as per protocol; we recorded the time-to-therapy as days since SAH diagnosis to FMT therapy, and outcomes as mortality at 30- and 90-days; Lille model at day 7 after FMT inception was used *per analogiam* with CS and with the kind consent of authors (responder to FMT by Lille model = score of 0.45 and less; non-responder to FMT = score above 0.45) [13]. Analysis of the gut microbiome by 16S RNA sequencing in donors and patients was recorded but not included in this analysis [37].

#### The exposure

After enrollment, patients were exposed to intervention — FMT (Table 1). We have adopted and modified for our healthcare context the original Sarin’s protocol [85]: each dose of 100 ml FMT was prepared from 30 g of stool from an unrelated healthy donor; donors were selected according to published criteria, including the SARS-CoV-2 update [86–88]. Fecal material was diluted with sterile saline, sieved, mixed with glycerol, and frozen at − 80 °C. Antibiotic pre-treatment was not part of the standard protocol. One hundred milliliters of freshly thawed FMT material was administered by a tube inserted via endoscope as distal to the duodenum as possible; intention-to-treat intervention was 5–7 doses over 7 days. Response to therapy was identical to the outcome defined above (Table 1).

**Table 1:** Baseline characteristics of patients with SAH, treated with FMT (*n* = 33^1^); Abbreviations: *ACLF* acute on chronic liver failure, *AD* acute decompensation of ACLD, *AHHS* Alcoholic Hepatitis Histological Score, *CRP* C-reactive protein, *CS* corticosteroids, *FMT* fecal microbial transplantation, *HVPG* hepatic venous pressure gradient, *MELD*, model for end stage liver disease, *NLR* neutrophil–lymphocyte ratio, SAH, severe alcoholic hepatitis, *TJB* transjugular liver biopsy; * = response to FMT

Age (years, mean)	49.7
Sex female, *n* (%)	11 (33.3%)
Non-responders to CS by Lille score—*n* (%)	28 (84.8%)
Non-eligible to corticosteroids, *n* (%)	5 (15.2%)
Time-to-FMT (from the admission to any hospital: door-to-syringe time, days)	24.75 (1–83)
MELD score (points, *p*)	28.66 (19–41)
MDF — Maddrey’s discriminant function (*p*)	73.75 (28–160)
AD without ACLF — number of patients, *n* (%)	6 (18.2%)
ACLF — number of patients, *n* (%)	27 (81.8%)
* ACLF 1*	*15 (45.5%)*
* ACLF 2*	*10 (30,3%)*
* ACLF 3*	*2 (6.1%)*
Lille model 7 days after FMT inception (response to FMT = 0.45 and less)	0.437 (0.006–0.988)*
CRP (0.00–5.00 mg/l)	39
NLR (1–3)	8.06
Liver frailty index (LFI) (mean) (frailty = LFI ≥ 4.5)	4.45
TJB performed (*n*, %)	15 (45.5%)
- AHHS	5.9
- Cirrhosis (*n*, %)	15 (45.5%)
- HVPG (mmHg, median)	15.1
FMT doses	5
30-day mortality (*n*, %)	5 (15.2%)
90-day mortality (*n*, %)	12 (36.4%)

^1^The primary cohort analyzed for associated factors consisted of 33 patients, while the cohort for comparison of mortality by propensity-score matching yielded 31 patients

### Statistical analysis

To compare 30-day and 90-day mortality in treated patients and controls, we used a control group of patients with SAH not treated by FMT from the registry of hospitalized patients RH7 (NCT04767945) [26]. To account for sizeable differences in age, sex, MELD, CRP, ACLF, bilirubin, and international normalized ratio (INR) between these two groups, we performed the nearest-neighbor propensity score matching with logit as the link function. We performed the statistical analysis using R and used the *matchit* function implementation from the MatchIt library. The balance measures were satisfactory, where the absolute standardized mean difference (ASMD) for MELD, CRP, Bilirubin, and INR was in the 0.1–0.2 range and ASMD for all the other predictors was below 0.1. Visual inspection of the distributions of propensity scores for control and treatment arms showed good alignment, so there appears to be no problem with the lack of support. As a sensitivity analysis, we performed full matching and optimal pair matching, and both these matching techniques led to qualitatively similar results. We used Kaplan–Meier curves to compare different groups of interest within FMT-treated patients, the reported *p* values are based on a log-rank test. *P* values < 0.05 are statistically significant [89–91].

For microbiome analysis and data processing, total DNA was extracted from 100 mg of stool using the QIAamp DNA Stool Mini Kit (Qiagen, Germany), following the manufacturer’s protocol, with an initial three rounds of homogenization using a FastPrep-24 5G homogenizer (MP Biomedicals, France). The V1–V3 region of 16S rDNA was amplified through 25 PCR cycles with 5xFIREPol MasterMix (Solis BioDyne, Tartu, Estonia). Post-verification via agar electrophoresis, a low-cycle indexing PCR was performed. The final libraries were purified using 1.8 × Agencourt AMPure XP beads (BeckmanCoulter, Brea, CA, USA). The quality and quantity of the sequencing libraries were validated with an Agilent 2100 (Agilent Technologies, Santa Clara, CA, USA) and a Qubit 2.0 Fluorometer (Thermo Fisher Scientific, Waltham, MA, USA). An equimolar pool of samples was sequenced on an Illumina MiSeq platform (Illumina, San Diego, CA, USA). After quality and length filtering using Trimmomatic [92], samples underwent quality assessment with FastQC [93] and were analyzed using the QIIME2 Core 2018.8.0 pipeline [94]. Taxonomic classification involved creating OTUs at 99% similarity through de novo clustering of features using search [95], followed by taxonomic assignment using a pre-trained naive Bayes classifier in the q2-feature-classifier QIIME2 plugin [96] which was trained on Silva 132 99% OTUs full-length sequences [97]. The beta diversity defined by unweighted Unifrac distance [98] was determined using scikit-bio and visualized as a PCoA plot in QIIME2.

## Results

**Fig. 1:**
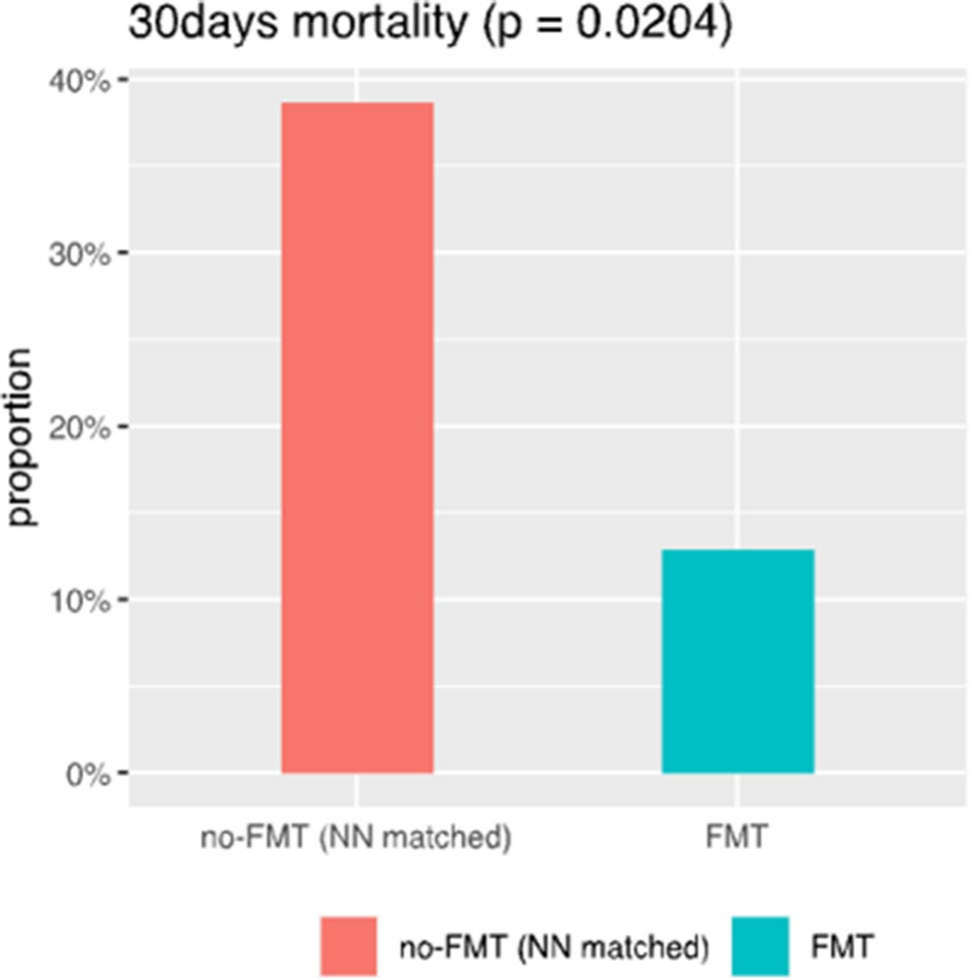
30-day mortality in the FMT group *(n* = *31)* as compared to the control group (*n* = 31). There is a statistically significant improvement in survival in the FMT group on day 30 (*p* = 0.0204)

**Fig. 2:**
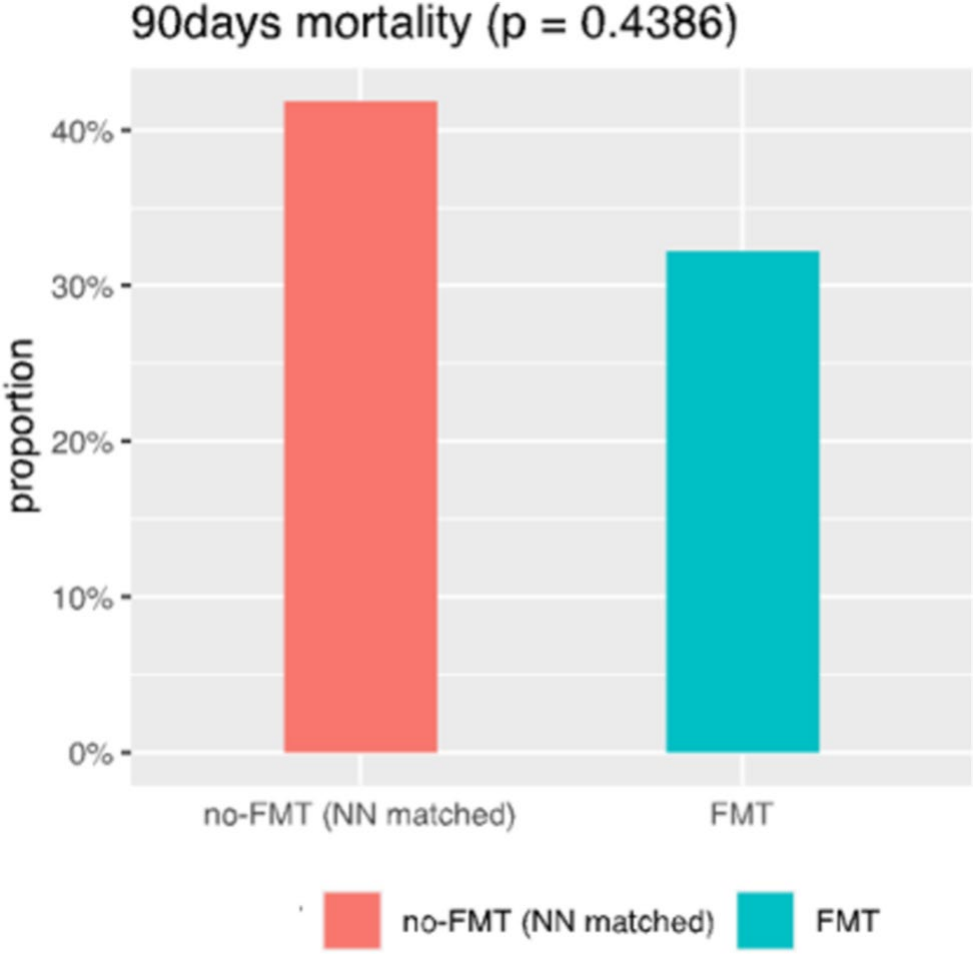
*90-day mortality in the FMT group (n* = *31****)**** vs*. the control group (n = 31). Survival in the FMT group on day 90 is better than in the control group, but the result is not statistically significant

In the active FMT arm, 44 patients with SAH were included by ITT, and 33 patients were analyzed per protocol after 11 had been excluded (patients who were administered less than 5 doses of FMT). Data from these 33 patients were used for the analyses inside the exposure cohort (outcome and associated factors). The mean age was 49.6 years; 11 patients (33.3%) were females. The median MELD score was 28.7, and ACLF was present in 27 patients (81.8%). The values of CRP and NLR were 39 mg/l (0.00–5.00 mg/l), and 8.06 (1–3), respectively. Fifteen patients (45.5%) underwent TJB, and the median HVPG and AHHS were 15.1 mmHg and 5.9, respectively. Thirty-day and 90-day mortality in the primary cohort of 33 patients was 15% and 36%, respectively. Of the 810 patients from RH7 identified for control-group selection, and after excluding patients with missing data for propensity-score matching, we had finally reached 273 and 31 observations for the control and treatment groups, respectively; therefore, for the case–control comparison of the outcome, we have used data from 31 (not 33) patients. After propensity score matching, FMT significantly improved 30-day mortality (38% vs. 12.5%, *p* = 0.0204) and non-significantly improved 90-day mortality (42% vs. 32%, *p* = 0.4386) (Table 2, Figs. 1, 2).

**Table 2:** Summarized statistics of the non-FMT (control) group (*n* = 273) and FMT group (*n* = 31)

FMT	no-FMT			FMT			Test
Variable	*N*	Mean	SD	*N*	Mean	SD	
Sex	273			31			X2 = 0.23
Male	168	61.5%		21	67.7%		
Female	105	38.5%		10	32.3%		
MELD	273	21.696	9.198	31	28.452	6.032	*F* = 15.919***
CRP	273	36.556	34.8	31	37.903	26.469	*F* = 0.044
ACLF_1D	273	0.67	0.896	31	1.194	0.833	*F* = 9.623***
ACLF_7D	273	0.747	1.053	31	0.935	0.854	*F* = 0.921
Bili_1D	273	202.746	202.15	31	406.806	176.41	*F* = 29.056***
Bili_7D	273	193.801	206.866	31	356.065	180.575	*F* = 17.543***
INR_1D	273	1.776	0.67	31	1.803	0.52	*F* = 0.049
INR_7D	273	1.814	0.829	31	1.757	0.494	*F* = 0.139
exitus 30	273	0.275	0.447	31	0.129	0.341	*F* = 3.083*
exitus 90	273	0.41	0.493	31	0.323	0.475	*F* = 0.887
Age	273	57.7	12.222	31	49.452	11.653	*F* = 12.795***

Statistical significance markers: *p < 01; **p < 0.05; *p* < 0.01.

We explored the explanatory power of different variables with univariate and multivariate logistic regressions to explore which variables were linked with a better prognosis within the FMT group. Also, we looked at the survival via the KM-curves (Supplementary Table 1–3 and supplementary Figs. 1–4).

**Fig. 3:**
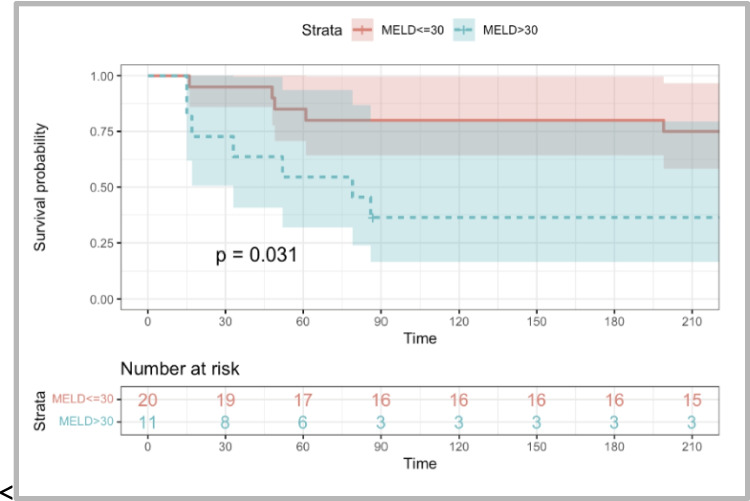
The Kaplan–Meier curve shows a comparison of survival rates within the FMT group. In this figure, patients were divided into two groups according to baseline MELD score. Patients with baseline MELD score < 30 had significantly better survival than patients with baseline MELD score ≥ 30 (*p* = 0.031)

**Fig. 4:**
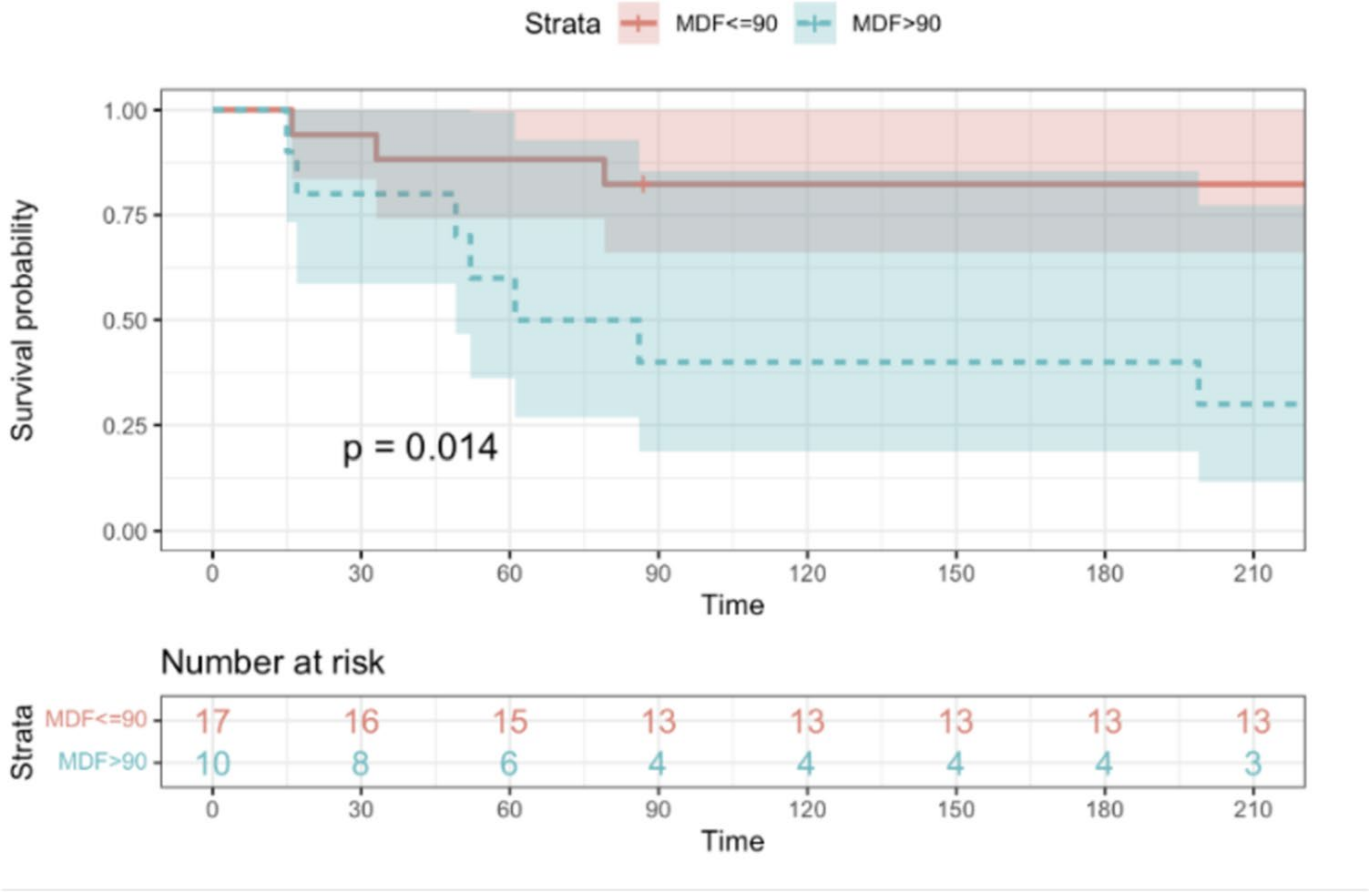
The Kaplan–Meier curve shows a comparison of survival rates within the FMT group. In this figure, patients were divided into two groups according to baseline MDF. Patients with baseline MDF < 90 had statistically better survival than patients with MDF ≥ 90 (*p* = 0.014)

**Fig. 5:**
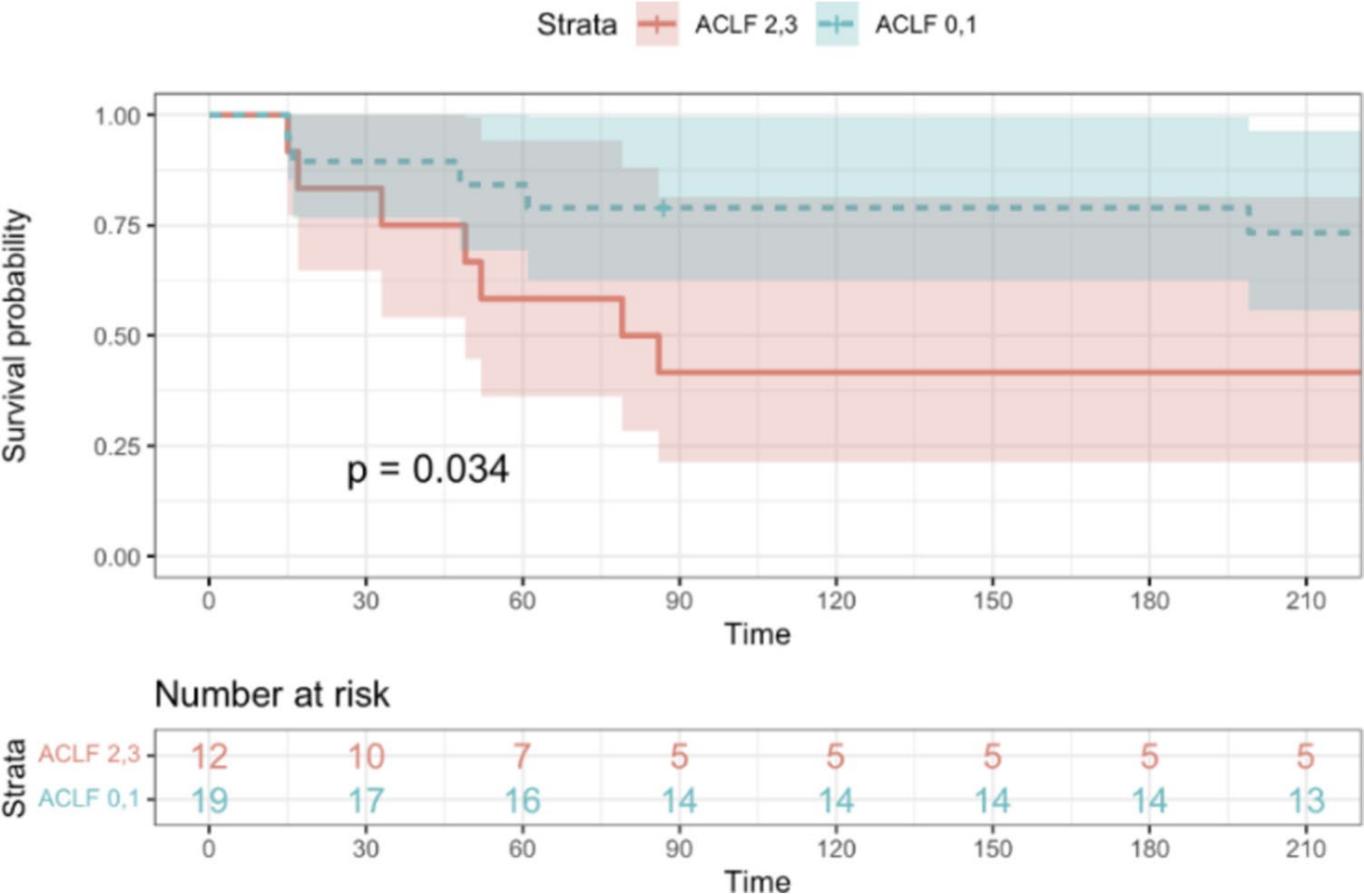
The Kaplan–Meier curve shows a comparison of survival rates within the FMT group. In this figure, patients were divided into groups according to baseline ACLF grade. Patients with baseline ACLF grades 0 and 1 had better survival than patients with baseline ACLF grades 2 and 3. This result was statistically significant (*p* = 0.034)

Both result clusters were then scrutinized for 3PM value: Univariate analysis identified MELD ≥ 30, MDF ≥ 90, and ACLF grade > 1 as significant predictors of 30-day mortality. Multivariate analysis was limited due to the sample size. Kaplan–Meier survival curves illustrated better survival in patients with three baseline variables: MELD < 30, MDF < 90, and ACLF grades 0–1. (Figs. 3, 4, 5).

### Microbiome analysis

**Fig. 6:**
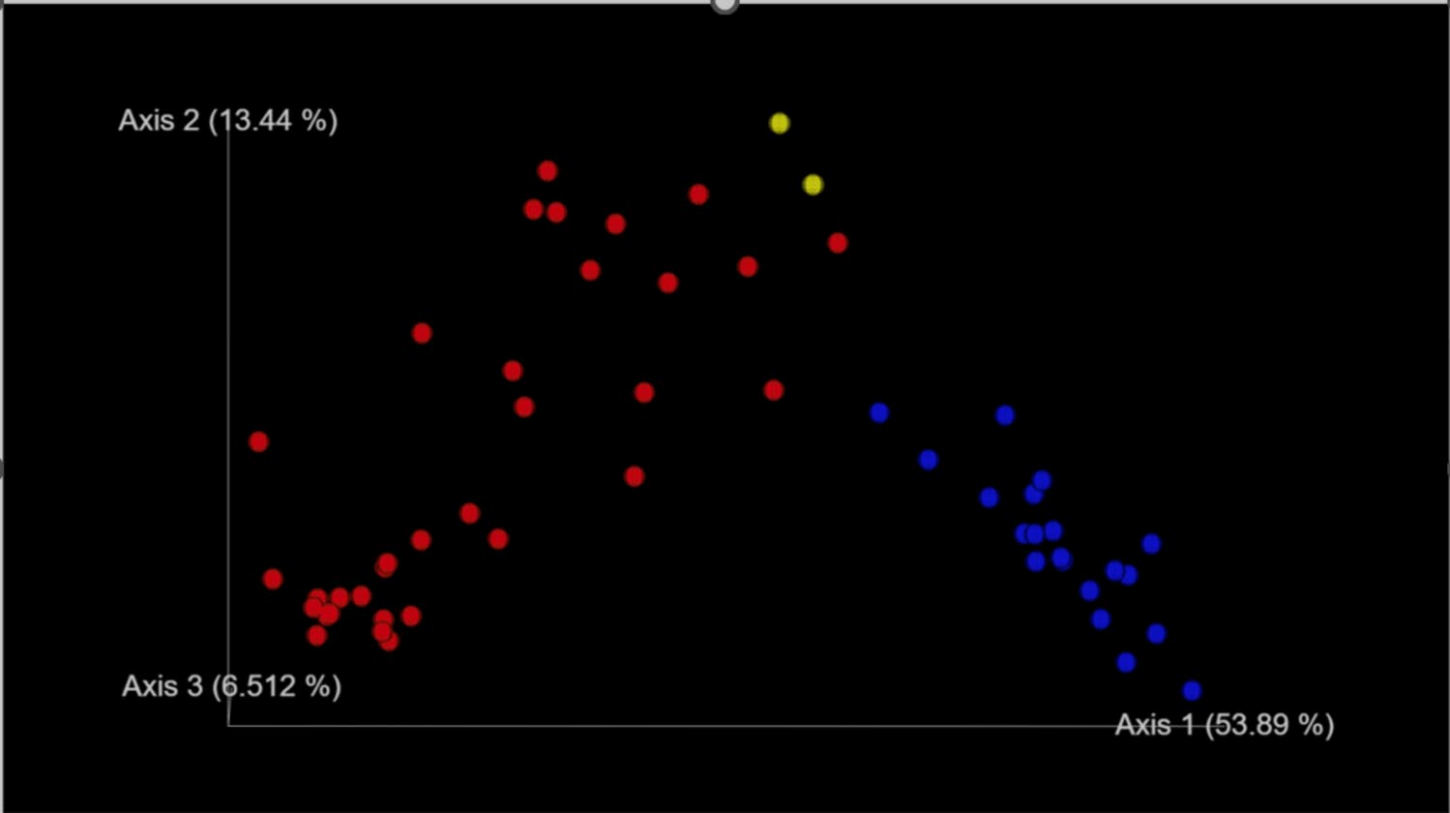
Gut microbiome diversity of patients with SAH treated with FMT (red dots), healthy individuals (blue dots), and donors (yellow dots). From this principal coordinate analysis plot, it is apparent that donors were closer to patients than to healthy controls (KŠ lab.)

Post-hoc microbiome analysis revealed significant differences between healthy controls, SAH patients, and donors. Dysbiosis patterns highlighted the potential for patient’s microbiome profiling in predicting FMT success (Fig. 6).

## Discussion and data interpretation

### The gut-liver *axis* as the therapeutic target

Effective therapy for patients with SAH beyond CS is an unmet need, which renders it the prime focus of interest to 3PM [99, 100]. Our study builds on and adds to the existing literature which proposes the gut-liver axis as the therapeutic target [35, 99, 101, 102]. The rationale behind addressing in our study the gut-liver axis was that (*i*) it is compliant with the current view of SAH pathophysiology, (*ii*) the regional healthcare system is permissive and disposes of crucial means, (*iii*) FMT, in general, has displayed a reassuring safety profile [103–106]. For the reasons delineated above, we decided to scrutinize FMT in SAH through the lens of 3PM template [31, 36, 60, 61, 73].

### The working hypothesis is verified towards the most difficult-to-treat NR/NE patients

Our results lend support to the notion that, in a real-life hepatology FMT in SAH is feasible and safe; moreover, it can improve individual outcomes of SAH patients who are the most difficult to treat — i.e., NR/NE. As hoped for in the hypothesis, 3PM scrutiny brought about cutoff values of readily available scores that significantly associated with individual patient outcomes and may enrich cohorts in future trials on both sides of the SAH phenotypes spectrum — on the futility and palliative care on the one hand, and trials on a novel pharmacotherapy, extracorporeal liver support systems, and early LT on the other [82]. The mortality effect of FMT against the propensity score-matched controls was significant at 30 days and not so at 3 months (Figs. 1, 2). We will discuss our results in light of similar studies, with an emphasis on a modest reduction of 90-day mortality, and with the view of the possible impact on future directions of 3PM research. *First*, it is not unusual for SAH that initial treatment success fades after the first month. For example, in the landmark STOPAH trial as well as in the subsequent meta-analyses of the effect of CS, mortality was significantly improved at 1 month but not later [13, 15, 18]. Taking into account the fact that our cohort was composed of the most difficult-to-treat NR/NE patients, in whom even a 1-month effect is very difficult to achieve, we consider the main outcome of the study to be positive. *Secondly*, our results should be interpreted in a wider context and to be compared to other studies with similar designs. Three studies from India on FMT in SAH have demonstrated various rates of early and long-term survival benefits (from 3 months to 3 years) [44, 80, 81, 107, 108]. In the first-of-the-kind study by the Sarin’s group, eight male NE patients with SAH, with a MELD score of 31 were administered fresh FMT prepared by a family member and administered over 7 days to duodenum. Markers of liver damage decreased promptly and improvement in microbiome composition and in outcome (later compared to historical controls) persisted for 1 year [86]. In the second study, Philips et al. compared FMT to either CS, nutrition, or pentoxifylline. Sixteen male patients allocated FMT fared significantly better at 3 months than responders to CS, and better than patients allocated nutrition or pentoxifylline. However, there was no statistically significant benefit at 1 month [80]. In the third study by Philips et al., with as of now the longest follow-up of 3 years, authors reported on 35 males with SAH, administered FMT from a healthy donor within 6 h of collection; the outcome was compared to 26 controls treated with standard of care (CS). Again, the study has shown the long-term benefit of FMT with a significantly improved 3-year survival (*p* = 0.0504). Of interest, Kaplan–Meier survival curves intersected, and the benefit was seen only after 3 months since FMT inception [81]. In the most recent, open-label study by Pande et al., authors compared 60 patients with SAH treated by CS to 60 patients treated with FMT. The authors demonstrated significant survival benefits at 90 days, but not at 30 days [108]. As seen from this analysis, despite the small number of studies, it is very difficult to compare results because of the heterogeneity of cohorts.

### Plausible explanations towards mitigated mortality

Several possible explanations exist for the waning mortality effect between 1 and 3 months, which can be roughly grouped into three areas: design-related, procedure-related, and patient-related. Considering the size of cohorts in studies claiming survival benefit in this interval, our study with 33 patients belongs to the medium-size category and we do not suppose type one error has accounted for the non-significant 3-month outcome. Despite some asymmetry in this direction, we believe that the propensity-score matched control group was not “too healthy,” which could have overshadowed the benefit of FMT. What could have explanatory potential, however, are the deviations from the originally published FMT protocols which we had to make; they concerned donor type, FMT procurement method, and cumulative FMT dose. Although we have adopted the original protocol described by Sarin et al., we could not comply in two important aspects: for logistical reasons, we were not able to use (1) freshly prepared (within 6 h) material from (2) relatives of patients. Instead, we made use of frozen material from the healthy unrelated donors which was otherwise handled according to the protocol described in the pivotal study [86]. Albeit these two factors could play a role in the outcome at 3 months, we do not consider them to be all-decisive. There is evidence stemming from studies on FMT in *Clostridioides difficile* infection suggesting that there are no substantial differences in outcome across the fresh-frozen and relative-unrelated donor dichotomies [44].

### Individualized patient profile and phenotyping as the key to improved individual outcomes

In our cohort, one donor-related factor that we consider substantial and possibly operative was the quality of FMT material according to the gut microbiome analysis (Fig. 6, yellow dots): donors displayed the principal coordinate analysis plot position outside the area of healthy controls; that could mean that FMT material was “suboptimal healthy.” Even this possibility is not certain, however, taking into account the controversy surrounding so-called enterotypes and using cluster boundaries as biomarkers.

Apart from the possible impact of FMT material, it is the domain of patients’ characteristics that lies the possible explanation of FMT’s suboptimal effect at 3 months. One is that the mortality in the control group at 3 months was better than we had expected for NR/NE patients [15, 18]. For this finding, we do not have an explanation other than precision in the bundle of care SAH at the liver unit due to the years-long focus on SAH research; albeit it remains the real possibility (and would be gratifying to us), we feel accepting this explanation would be premature, a.o., because it would be at odds with the literature which has not shown improving prognosis in patients with ALD syndromes over time. Therefore, in accordance with the latest study by Philips et al., we suspect other factors uninvestigated in this study and incorporated in the pathophysiology of SAH might be at play: certain gut microbiome taxa, cytolysin production and other constituents of the microbiome, bile acids, mitochondrial stress, etc. [82]. For these factors, we plan to cover in the design of the forthcoming prospective study. Anyhow, the question will remain whether there is a value in improving individual patient outcomes at “only” 30 days (and not later). Before the necessary formal cost-effectiveness analyses take place, we are convinced that opening the new 1-month window of opportunity in critically ill patients with SAH NR/NE (usually doomed) would provide the room for re-considered and re-communicated palliative care on the one hand or, for re-considered radical intent as early LT or experimental therapies.

As of now, we are not able to explain why frailty, portal hypertension, and histological activity have not came out as predictors of individual patient outcomes; although such unexpected results are usually fertile ground for new hypotheses, we would like to postpone scientific speculation until substantially more patients are enrolled. Considering increasing SAH prevalence, these open questions have to be answered soon and followed by cost-effective mitigation measures tailored to individualized patient profiles.

### Limitations

The primary limitations include sample size in the exposure arm, retrospectively accrued control group (albeit from the large dataset), and the absence of the taxa-by-taxa pre-FMT microbiome analysis in both donors and recipients. Not all the analyzed patients have had HVPG measured; hence, the absence of its association with the outcome will have to be corroborated in a larger cohort that is underway; the same applies to the liver histology. Another limitation is related to histology: we have not yet paired clinical NIAAA diagnostic criteria for SAH with the Altamirano index. Therefore, the current analysis of our cohort should be seen as “probable SAH diagnosis,” which is the case in the majority of trials on SAH up to now [24]. Further limitation is that we have not included in recorded variables continued alcohol abuse (or sobriety, for that matter). Considering that abstinence is the strong predictor of prognosis — albeit usually materialized beyond the 3 months which was our follow-up interval — this shortcoming cannot be excluded as the possible confounder weakening effect of FMT at 90 days. Even though patients spent a substantial part of the follow-up period in the hospital and after discharge remained in a seriously deteriorated state not conducive to drinking — without the due evidence we cannot be sure that a relapse to alcohol abuse has not interfered with our results at 90 days. We plan to amend this limitation in the forthcoming phase of research.

## Implication for predictive, preventive, and personalized approach

First and foremost, our study highlights the importance of the methodological and conceptual role that 3PM template may play in the search for both the novel therapies and for improving the existing standard of care. Continued adherence to the 3PM methodology may stand behind both the improved 30-day survival in the FMT group as well as the relatively low 90-day mortality in the control group which could paradoxically be one of the reasons leading to the non-significant FMT effect after 3 months. Such a positive, double-impact of the 3PM concept on the individual patient outcomes in both exposure and control groups fully expresses its potential in the preventive domain of 3PM. Second, 3PM-guided analysis has identified factors predictive of suboptimal therapeutic effects and poor individual outcomes. Albeit the predictive power of baseline MELD ≥ 30, MDF ≥ 90, and ACLF grade > 1 should be confirmed in further research, they concur with the guidelines on the proposed methodology of AH/SAH studies [109–111]; predicted futility of intervention will allow for the exclusion of patients not expected to respond from randomized controlled trials and enrich other cohorts. This “prevention of suboptimal therapeutic effect” will ensure a more personalized allocation of experimental therapies and will provide an evidence-based framework for palliative care research. In our clinical practice and research, results of baseline MELD, MDF, and ACLF scores predictive of non-response to FMT will help us to allocate patients alternative therapies or palliative care and, together with the more foresighted selection of donors for FMT, to hope for improved long-term outcomes in the FMT group [112]. This would close the full first-level circle of the 3PM spiral of prevention, prediction, and personalization in the SAH arena.

## Outlook from the 3PM perspective

Based on the ample evidence, we expect increasing global research activity across the spectrum of phenotypes of ALD including SAH. In this line, since the 3PM methodology is the perfect match for investigating such a complex problem as ALD–spanning AUD, epidemiology in motion, public health with extremely difficult-to-implement preventive policy measures, stigma, inequalities in health, suboptimal health, chronic lack of interest in funding research, complex pathophysiology, and experimental and real-life clinical research, we expect increasing interest in *mysterium conjunction* between ALD, SAH, and 3PM methodology. We humbly consider our work to be an example of how advantageous this approach might be. In the narrow domain of FMT in SAH, we expect the following evolution in the field: *P1. Optimization of preventive strategies.* We expect research on new methods to better understand ALD-specific gut dysbiosis which will be levered as a preventive-therapeutic target; explored in this regard will be prebiotics, probiotics, postbiotics, phages, dietary interventions, and other approaches [34, 35, 37, 38]. *P2. Refinement of predictive tools:* Moving forward preventive domain of 3PM is impossible to conceive without more precise phenotyping of patients at risk; therefore, we expect activities in developing more accurate prognostic models, incorporating psychology, genetics, microbiome, and mitochondria, and other clinical and laboratory data to better identify the ideal candidate for FMT (or patients bound to fail FMT). To enhance accuracy in predicting treatment response and outcome, advanced analytics, machine learning, and other tools from the realm of artificial intelligence will be introduced [34, 39–41]. *P3. Advances in personalization:* Personalized FMT protocols based on comprehensive patient profiling overlap with the domain of prediction. Precisely tailored FMT donor selection criteria will be developed to maximize treatment efficacy and minimize adverse effects [42]. One of the possible directions will be the patient-specific dysbiosis-directed FMT or its variant (e.g., phage therapy). *Long-term outcome:* As they are scarce, long-term studies will be conducted to evaluate the minimal duration of FMT to ascertain its sustained effects on liver function and patient survival. Assessed will be the potential of FMT to improve patient-reported outcomes such as disease-related quality of life, and these measures will complement the next-generation 3PM metrics of FMT outcome. *Integration with other therapies:* It is necessary to investigate the combination of gut dysbiosis-targeted therapies with other established and investigational therapeutic modalities focused on psychotherapy of craving, pharmacotherapy targeting immunity, liver regeneration, mitochondrial stress, and others. Investigated will be combinations with synergistic effects to enhance treatment response in SAH patients.

## Conclusions and expert recommendations

In the most difficult-to-treat cohort of patients with SAH—NA/NR, FMT improved 30-day mortality. Pre-treatment factors associated with improved individual outcomes included MELD ≤ 30, MDF ≤ 90, and ACLF < 2 — which allow for drawing a more precise 3PM management flowchart. Our results lend support to the notion that, for targeting gut microbiome by research and intervention, the 3PM approach is the appropriate template for ascertaining maximal effectiveness in achieving (i) *personalized phenotyping and stratification of the patients with SAH* for the clinical research and practice; (ii) *multilevel predictive diagnosis* related to personalized treatment allocation; (iii) *prevention of futile and sub-optimally effective therapy;* as well as (iv) *targeted prevention* of poor individual outcome. Our results add to the research along the pathogenetic pathways of SAH, which have investigated mechanisms behind the diverse susceptibility to the effect of alcohol, host-specific variability of systemic inflammation, and the role of the gut microbiota thereof. At this moment, however, it seems appropriate to not only delve deeper into the gut-liver axis but to also focus on the other drivers of the alcohol abuse-SAH-ACLF-poor outcome continuum, e.g., on the role of the mitochondrial health, hepatocyte regeneration, metabolism of alcohol, and immuno-pathogenesis of SAH.

## Supplementary Information

Below is the link to the electronic supplementary material.

Supplementary file1 (DOCX 528 KB)

## Data Availability

No datasets were generated or analysed during the current study.

## Abbreviations

3PM: Predictive, Preventive, and Personalized Medicine
ACLD: Advanced chronic liver disease
ACLF: Acute-on-chronic liver failure
AHHS: Alcoholic Hepatitis Histological Score
ALD: Alcohol-associated liver disease
ALT: Alanine aminotransferase
AST: Aspartate aminotransferase
ATP: Adenosine triphosphate
AUDIT: Alcohol Use Disorders Identification Test
CASP4: Caspase-4
CASP11: Caspase-11
CRP: C-reactive protein
CS: Corticosteroids
DAMPs: Damage-associated molecular pattern molecules
DNA: Deoxyribonucleic acid
EASL/EF CLIF: European Association for the Study of the Liver/ European Foundation for the Study of Chronic Liver Failure
FMT: Fecal microbiota transplantation
GSDMD: Gasdermin D
HCC: Hepatocellular carcinoma
HVPG: Hepatic venous portal gradient
IL-1β: Interleukin—1 beta
IL-6: Interleukin – 6
INR: International normalized ratio
ITT: Intention-to-treat
LFI: Liver Frailty Index
LPS: Lipopolysaccharide
LT: Liver transplantation
MELD: Model for end-stage liver disease
MDF: Maddrey’s Discriminant Function
NE: Non-eligible
NIAAA: The National Institute on Alcohol Abuse and Alcoholism
NLR: Neutrophil to lymphocyte ratio
NR: Non-responders
PAMPs: Pathogen-associated molecular patterns
PRRs: Pattern-recognition receptors
RH7: Registry HEGITO 7
SARS-COV 2: Severe acute respiratory syndrome coronavirus 2
SAH: Severe alcohol-associated hepatitis
SIBO: Small intestinal bacterial overgrowth
SIRS: Systemic inflammatory response syndrome
STOPAH: Study-Steroids or pentoxifylline for alcoholic hepatitis
TJB: Transjugular liver biopsy
TLRs: Toll-like receptors
TNF: Tumor necrosis factor
