# Supplementary material for: 3PM-guided innovation in treatments of severe alcohol-associated hepatitis utilizing fecal microbiota transplantation
Source: EPMA J. 2024 Oct 31;15(4):677–92. doi: 10.1007/s13167-024-00381-5 (PMC11612130; doi:10.1007/s13167-024-00381-5)
Supplement: Supplementary file 1 — Supplementary file1 (DOCX 528 KB) [file 13167_2024_381_MOESM1_ESM.docx]

**Supplementary materials**


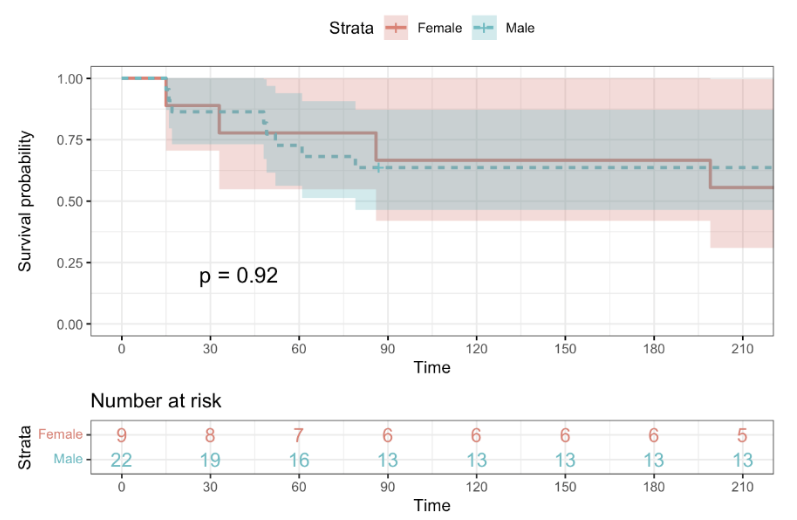


**S Figure 1**. The Kaplan-Meier curve shows a comparison of survival rates within the FMT group. In this figure, patients were divided into groups according to gender (male/female). We did not observe any statistical difference in survival between these groups. (p=0.92)


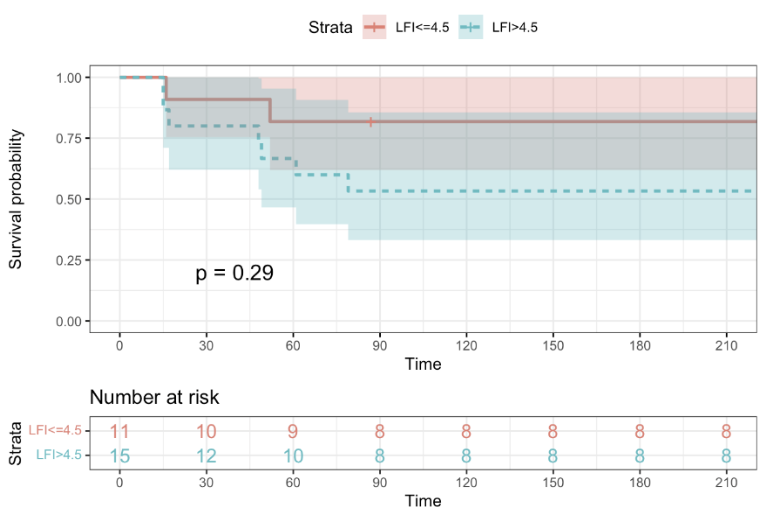


**S Figure 2. The** Kaplan-Meier curve shows a comparison of survival rates within the FMT group. In this figure, patients were divided into groups according to liver frailty index (LFI) measured at baseline. The value of LFI ≥ 4.5 defines the frail stage of the patient, values that are below this cut-off define pre-frail and robust patients. We did not observe any statistical difference in survival between these groups. (p=0.29).


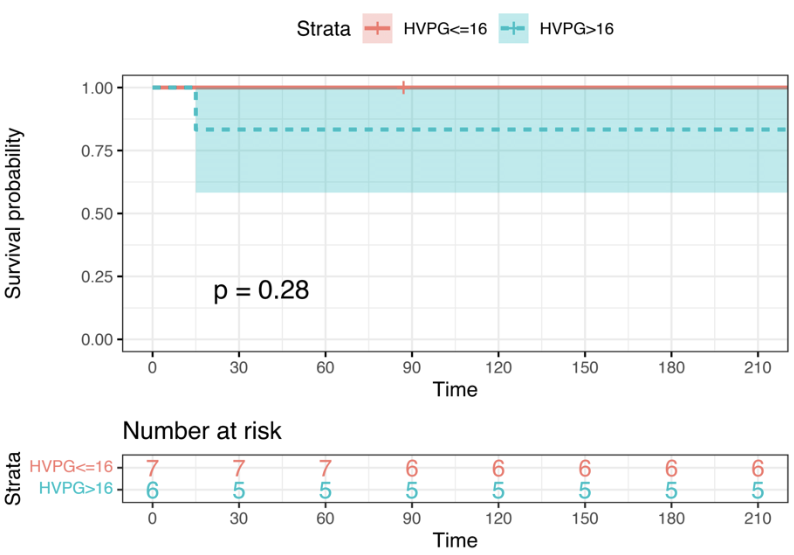


**S Figure 3. The** Kaplan-Meier curve shows a comparison of survival rates within the FMT group. In this figure, patients were divided into groups according to hepatic venous pressure gradient (HVPG) measured at baseline. The value of HVPG ≥ 16 mmHg defines severe portal hypertension. We did not observe any statistical difference in survival between these groups. (p=0.28)


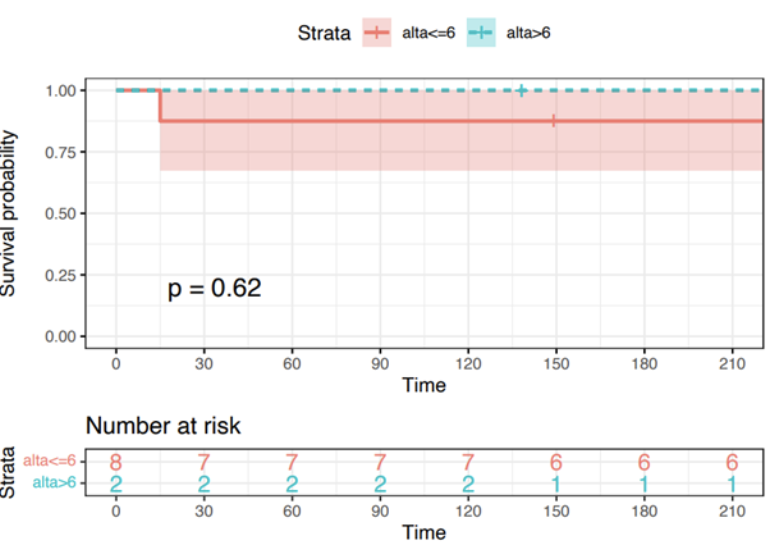


**S Figure 4.** The Kaplan-Meier curve shows a comparison of survival rates within the FMT group. In this figure, patients were divided into groups according to the Alcoholic Hepatitis Histological Score (AAHS) calculated by pathologists from samples obtained by transjugular liver biopsy at baseline. The value of AAHS ≥ 6 defines severe alcoholic hepatitis and is associated with a bad prognosis. We did not observe any statistical difference in survival between these groups. (p=0.62)

**S Table 1.** Unifactorial analysis of factors associated with 30-day mortality within FMT group. The only significant factor was the MELD score.


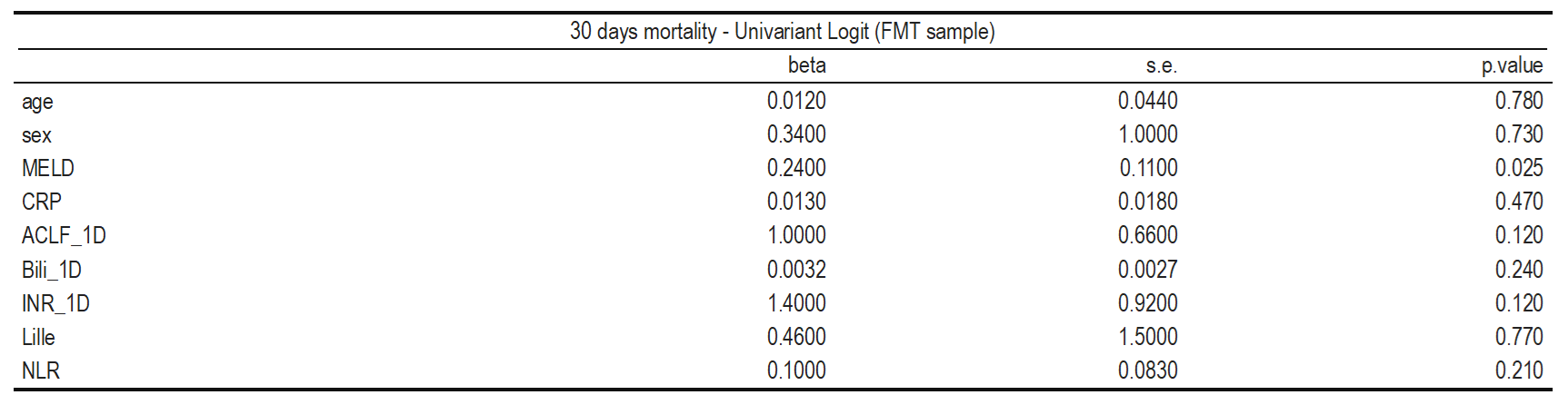


**S Table 2.** Unifactorial analysis of factors associated with 90-day mortality within FMT group. The significant factors were MELD score and INR measured at baseline.


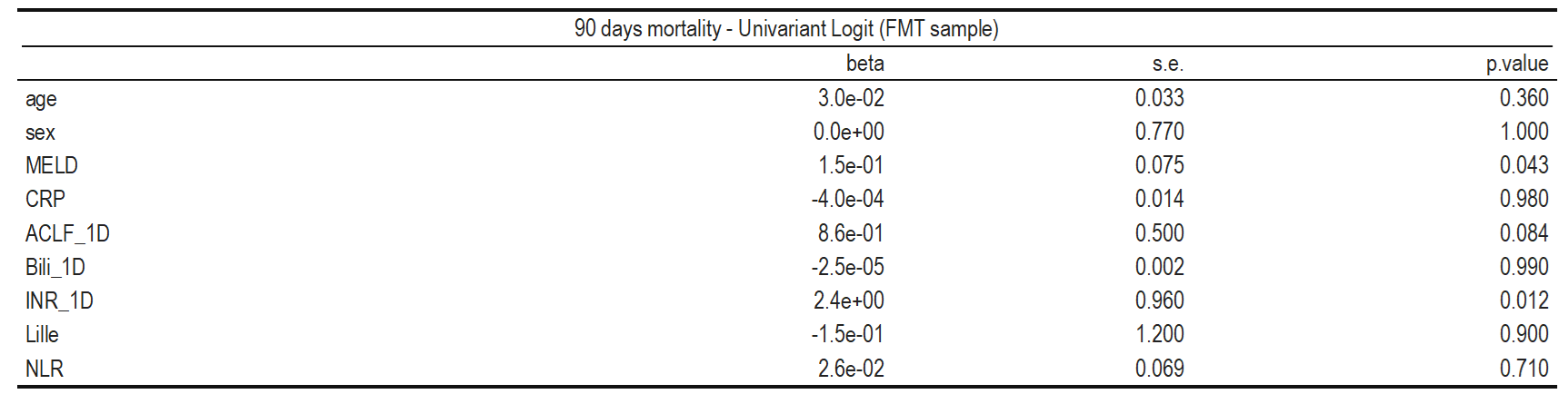


**S Table 3.** Multifactorial analysis of factors associated with 30-day and 90-day mortality within FMT group. We confirmed the MELD score as a factor associated with 30-day mortality. Bilirubin and INR measured at baseline were associated with 90-day mortality

**
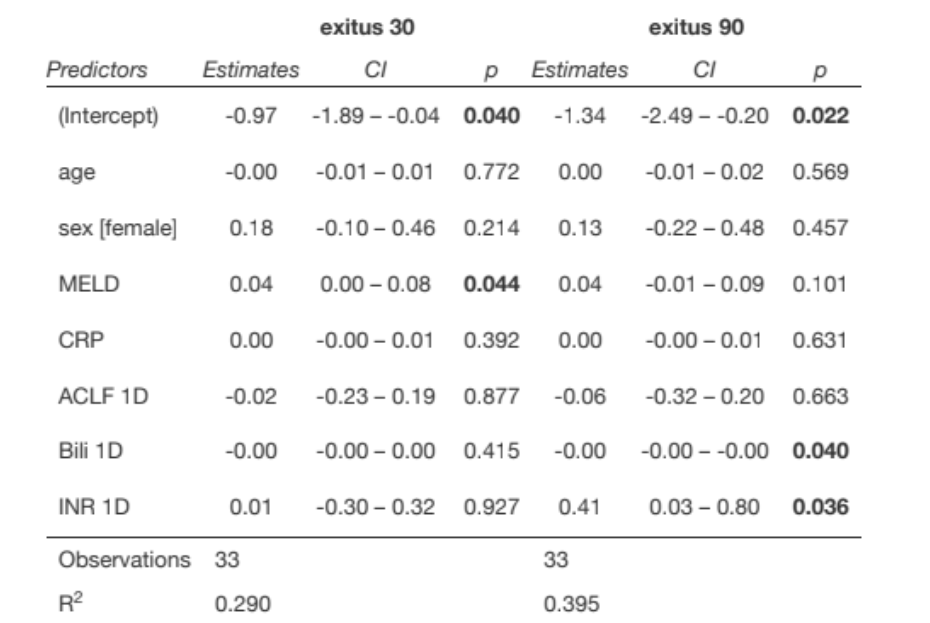
**
